# Infective Endocarditis Caused by Streptococcus viridans in a Previously Healthy Man: A Case Report

**DOI:** 10.7759/cureus.76896

**Published:** 2025-01-04

**Authors:** Sara Fontaínhas, Bárbara Baptista, Maria I Bertão, Beatriz Lima, Ricardo Gomes

**Affiliations:** 1 Internal Medicine, Hospital Distrital da Figueira da Foz, Figueira da Foz, PRT; 2 Internal Medicine, Centro Hospitalar Universitário Cova da Beira, Covilhã, PRT

**Keywords:** bicuspid aortic valve, cardiac vegetations, congenital aortic valvulopathy, infective endocarditis, streptococcus viridans

## Abstract

Infective endocarditis (IE) is a rare and potentially fatal infection of the heart valves, often caused by *streptococci, staphylococci, and enterococci.* This case is particularly significant as it describes IE caused by *Streptococcus viridans* in a 36-year-old male patient, previously healthy with no known risk factors, who presented with a three-week history of fever, night sweats, and fatigue. Initially, the clinical presentation was attributed to an atypical infection due to his occupational exposure, and he was empirically treated with doxycycline, showing apparent clinical improvement. However, blood cultures were positive for *Streptococcus viridans*, leading to hospitalization and suspicion of endocarditis.

Echocardiography revealed congenital aortic valvulopathy (bicuspid aortic valve) with moderate regurgitation but no evidence of vegetation. A transesophageal echocardiogram identified vegetations, confirming the diagnosis of IE. Treatment with ceftriaxone and gentamicin resulted in a favorable clinical response after four weeks.

This case highlights the diagnostic challenges of IE in atypical presentations and the importance of considering IE in patients with fever and positive blood cultures, even in the absence of known cardiac disease or previous risk factors. It also emphasizes the need for timely diagnosis to prevent severe complications associated with endocarditis. The authors aim to underscore the indolent course of IE in this rare clinical presentation.

## Introduction

Infective endocarditis (IE) is a rare but potentially life-threatening infection of the inner layer of the heart, most commonly affecting the heart valves [1]. The incidence of IE ranges from 3 to 10 cases per 100,000 person-years [2], with higher rates in men and older adults [3]. Common causative organisms include *streptococci, staphylococci, and enterococci* [4]. Diagnosing IE is challenging due to its nonspecific symptoms, such as fever, chills, night sweats, fatigue, and weight loss, which can mimic other conditions. Clinical suspicion is particularly low in patients without clear risk factors like prosthetic valves, congenital heart disease, or intravenous drug use. Initial tests, such as transthoracic echocardiography, may fail to reveal conclusive findings, and more sensitive diagnostic tools, such as transesophageal echocardiography or repeated blood cultures, are often required, delaying diagnosis and treatment. These delays increase the risk of complications, including embolic events or severe valve damage.

In this case report, we present a 36-year-old previously healthy man with no known risk factors, who presented with a febrile syndrome and fatigue lasting three weeks. His diagnosis of IE caused by *Streptococcus viridans *was initially challenging, as there were no evident cardiac manifestations such as murmurs or heart failure. The definitive diagnosis was made only after transesophageal echocardiography, which revealed a congenital aortic valve abnormality (bicuspid valve), the first clinical indication of a previously undiagnosed valvulopathy. This case highlights the diagnostic challenge in patients with rare presentations of IE, particularly when there are no evident risk factors.

Treatment of IE involves prolonged intravenous antibiotic therapy, which can be complicated by bacterial resistance. Patients with valvulopathies, like congenital bicuspid valves, are at increased risk for recurrent infections, underscoring the importance of long-term follow-up. This case contributes to the literature by addressing the limited data on IE caused by *Streptococcus viridans* in patients with undiagnosed congenital valvulopathies, offering valuable insights into the management of rare presentations of IE.

## Case presentation

The patient was a 36-year-old, previously healthy male with no underlying immunological conditions or heart disease, no history of previous dental or surgical procedures, and no history of prior endocarditis. He was also not an intravenous drug user or a regular alcohol consumer. He presented to the emergency department with a three-week history of fever, night sweats, and persistent fatigue. The patient resided in France and worked in a meat processing factory, where he had exposure to zoonotic pathogens or environmental risks. No family history was noted, and he had no known allergies or regular medications.

On physical examination, the patient was febrile with a temperature of 38.5 °C. His oral hygiene was good, with no visible dental caries. Cardiac auscultation revealed no audible murmurs. There were no peripheral stigmata of infective endocarditis such as Janeway lesions, nailbed hemorrhages, or Osler nodes. His joints were generally painless and non-tender.

Complementary exams, including a complete blood count, biochemistry, viral serologies, urinalysis, chest radiograph, and abdominal and renal ultrasound, showed no significant abnormalities. Given the suspicion of a zoonotic infection or infection caused by atypical community-acquired organisms, doxycycline was empirically initiated.

The patient was re-evaluated four days later. Upon reassessment, the patient reported clinical improvement, with sustained apyrexia for more than two days. However, due to the presence of positive blood cultures for *Streptococcus viridans*, he was hospitalized for further investigation of bacterial endocarditis.

**Figure 1:**
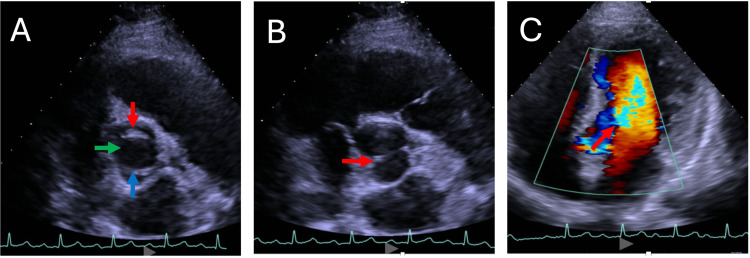
Bicuspid aortic valve with anteroposterior cusps (Sievers type 0) (A): Aortic valve opening during systole, showing two leaflets in a bicuspid configuration. The red arrow indicates the anterior cusp, the blue arrow points to the posterior cusp, and the green arrow highlights the aortic valve opening. (B): Aortic valve during diastole, demonstrating the presence of two leaflets in a bicuspid configuration. The red arrow highlights the raphe. (C): Color Doppler imaging illustrating a moderate aortic regurgitation jet (red arrow), consistent with altered hemodynamics through the bicuspid aortic valve.

A transthoracic echocardiogram was performed upon admission, which revealed the presence of congenital aortic valve disease - aortic bicuspidia - with apparently moderate regurgitation (Figure 1). No vegetation was observed.

**Figure 2:**
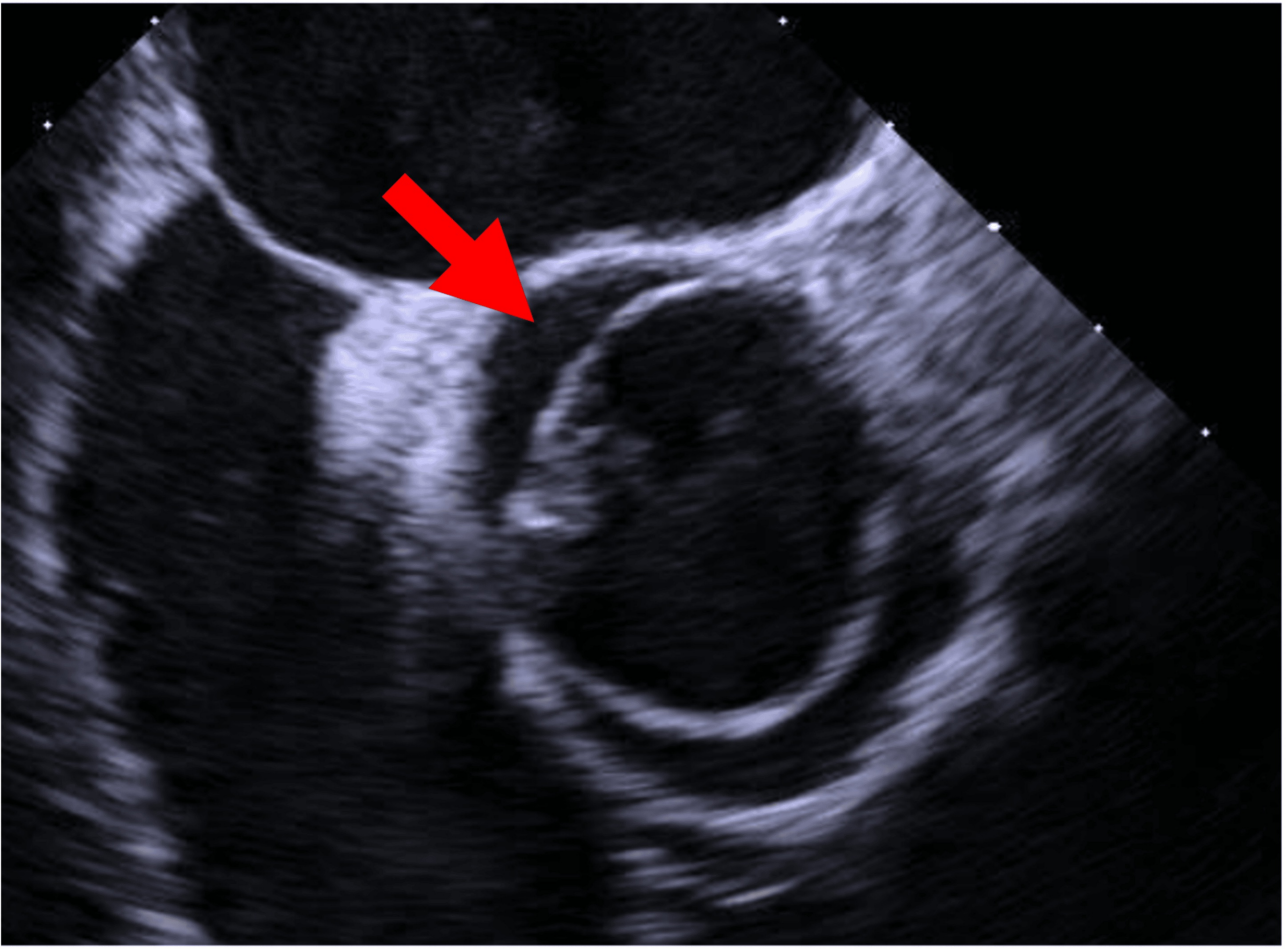
Bicuspid aortic valve The arrow indicates the fusion of the cusps in the aortic valve, a hallmark of bicuspid aortic valve morphology.

**Figure 3:**
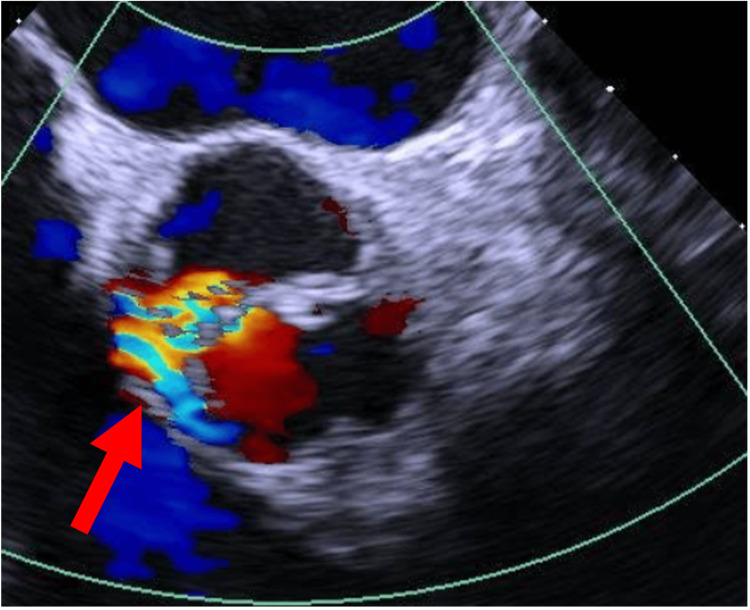
Apparently moderate-grade maximum regurgitation The arrow highlights the aortic regurgitation jet, visualized on color Doppler, indicating high-velocity retrograde blood flow through the aortic valve during diastole.

The case was discussed with the cardiology team and the diagnosis of endocarditis was assumed. A transesophageal echocardiogram was scheduled, which revealed the presence of congenital aortic valve disease (bicuspid aortic valve) (Figure 2), with apparently moderate regurgitation (Figure 3). A hyperechoic, filamentous structure with vibratory motion was observed only at the base of the coronary cusp on its aortic surface, with results suggestive of vegetation. According to the Duke criteria, the diagnosis of endocarditis was considered definitive, as the patient presented: two major criteria (identification of the pathogen in blood cultures and vegetation identified on transesophageal echocardiogram) and two minor criteria (fever and aortic valve disease).

The patient completed a four-week empirical antibiotic regimen with ceftriaxone and gentamicin. The susceptibility results for *Streptococcus mitis* are presented in Table 1. He showed good clinical and analytical progress and was discharged with guidance for cardiology consultations.

**Table 1:** Susceptibility results for Streptococcus mitis (viridans streptococcus)

Antibiotics	MIC (mg/L)	Susceptibility
Ampicillin	≤0.25	Sensitive
Cefotaxime	≤0.2	Sensitive
Ceftriaxone	=0.25	Sensitive
Clindamycin	≤0.25	Sensitive
Meropenem	N/A	Sensitive
Penicillin-G	=0.25	Sensitive
Teicoplanin	≤0.12	Sensitive
Vancomycin	=0.25	Sensitive

During the cardiology follow-up consultation, a new electrocardiogram and transthoracic echocardiogram were performed. One year after the episode of infective endocarditis, the electrocardiogram showed sinus rhythm with a first-degree atrioventricular block. The echocardiogram revealed a mildly dilated left ventricle with an ejection fraction (EF) of 60% and moderate to severe aortic insufficiency. These cardiac alterations may be considered as potential sequelae of infective endocarditis. Additionally, the patient was diagnosed with arterial hypertension, most likely secondary to aortic insufficiency.

## Discussion

This case highlights the importance of considering bacterial endocarditis in the differential diagnosis of patients with positive blood cultures and fever, even in the absence of apparent vegetation on echocardiography [5]. The absence of vegetation in the initial echocardiographic evaluation makes this diagnosis challenging. In this case, it was the identification of a microorganism typical of endocarditis, *Streptococcus viridans*, which increased the clinical suspicion of this condition, even though typical sources of this agent were excluded. Due to its higher sensitivity, transesophageal echocardiography is often necessary to confirm the presence of vegetation. The presence of congenital aortic valvulopathy in this patient was a likely predisposing factor for the development of endocarditis [6]. The patient's good clinical and analytical response to antibiotic therapy is consistent with previous reports of successful treatment of *Streptococcus viridans* endocarditis with ceftriaxone and gentamicin [7].

The multidisciplinary approach, "The endocarditis team" (including cardiology, infectious diseases, and cardiac surgery teams), is crucial for the management of complex IE cases. In fact, IE can progress to complications such as heart failure, uncontrolled infection with abscesses, false aneurysms, fistulas, and an increased risk of embolization. In this case, the patient progressed with worsening aortic insufficiency and even arrhythmia. Regular follow-up and monitoring of valvular function in patients with congenital valvular abnormalities are essential to establish a more appropriate therapeutic plan. In the reported case, subacute endocarditis caused by *Streptococcus viridans* is described, which is typically associated with a better prognosis and low mortality, especially when diagnosed early and in the absence of complications.

## Conclusions

In conclusion, we report a case of infective endocarditis caused by *Streptococcus viridans* in a 36-year-old, previously healthy man. According to the current Duke criteria, this patient presented two major criteria and two minor criteria, which establishes the diagnosis of endocarditis as definitive. This case highlights the importance of considering this entity in the differential diagnosis of young patients with positive blood cultures and fever, even in the absence of apparent vegetation on transthoracic echocardiography. As evident in this clinical case, transesophageal echocardiography is critical in cases with negative transthoracic echocardiography results. This fact is particularly apparent when there are risk factors or persistent bacteremia. The patient’s good clinical and analytical response to antibiotic therapy underscores the importance of early diagnosis and treatment of this potentially life-threatening infection.

Through this case, the authors aim to emphasize the importance of considering the diagnosis of endocarditis even in young patients without apparent risk factors, reinforcing the need for performing transesophageal echocardiography in patients with positive blood cultures for typical pathogens. Additionally, it is important to highlight that only through transthoracic echocardiography was a moderate degree of congenital valvulopathy identified. Although previously asymptomatic, it represents a subtle risk factor for endocarditis, even in asymptomatic patients, requiring regular surveillance by cardiology.
